# White matter abnormalities and multivariate pattern analysis in anti-NMDA receptor encephalitis

**DOI:** 10.3389/fpsyt.2022.997758

**Published:** 2022-09-23

**Authors:** Shengyu Yang, Ying Wu, Lanfeng Sun, Meigang Ma, Sijie Ou, Youshi Meng, Jie Meng, Chunmei Zeng, Qi Huang, Yuan Wu

**Affiliations:** Department of Neurology, The First Affiliated Hospital of Guangxi Medical University, Nanning, China

**Keywords:** white matter, anti-NMDA receptor encephalitis, multivariate pattern analysis, tract-based spatial statistics, MRI

## Abstract

**Objective:**

This study aimed to investigate white matter (WM) microstructural alterations and their relationship correlation with disease severity in anti-NMDA receptor (NMDAR) encephalitis. Multivariate pattern analysis (MVPA) was applied to discriminate between patients and healthy controls and explore potential imaging biomarkers.

**Methods:**

Thirty-two patients with anti-NMDAR encephalitis and 26 matched healthy controls underwent diffusion tensor imaging. Tract-based spatial statistics and atlas-based analysis were used to determine WM microstructural alterations between the two groups. MVPA, based on a machine-learning algorithm, was applied to classify patients and healthy controls.

**Results:**

Patients exhibited significantly reduced fractional anisotropy in the corpus callosum, fornix, cingulum, anterior limb of the internal capsule, and corona radiata. Moreover, mean diffusivity was increased in the anterior corona radiata and body of the corpus callosum. On the other hand, radial diffusivity was increased in the anterior limb of the internal capsule, cingulum, corpus callosum, corona radiata, and fornix. WM changes in the cingulum, fornix, and retrolenticular part of the internal capsule were correlated with disease severity. The accuracy, sensitivity, and specificity of fractional anisotropy-based classification were each 78.33%, while they were 67.71, 65.83, and 70% for radial diffusivity.

**Conclusion:**

Widespread WM lesions were detected in anti-NMDAR encephalitis. The correlation between WM abnormalities and disease severity suggests that these alterations may serve a key role in the pathophysiological mechanisms of anti-NMDAR encephalitis. The combination of tract-based spatial statistics and MVPA may provide more specific and complementary information at the group and individual levels.

## Introduction

Anti-NMDA receptor (NMDAR) encephalitis is the most frequent autoimmune encephalitis, first described by Dalmau in 2007 (1). Its clinical symptoms are complex and varied, often manifesting with aberrant psychiatric behavior, seizures, decreased consciousness, motor impairment, language impairment, dysautonomia, or central hypoventilation (2, 3). Despite the severe clinical presentation of anti-NMDAR encephalitis, clinical routine brain MRI is unremarkable in most patients (4). Furthermore, even in patients with abnormal conventional MRI findings, these abnormalities are typically subtle and non-specific. Therefore, there are limitations in the diagnostic and prognostic assessment of conventional MRI in anti-NMDAR encephalitis.

Advanced structural and functional MRI techniques can provide additional information about the disease compared to conventional MRI and are important for the diagnosis and prognosis of neurological disorders (5, 6). Although their application in anti-NMDAR encephalitis is gaining attention, studies on structural and functional MRI of anti-NMDAR encephalitis are scarce. A study has indicated that reduced bilateral hippocampus functional connectivity and impaired white matter (WM) integrity in patients with anti-NMDAR encephalitis, most notably reduced fractional anisotropy (FA) values in the cingulate gyrus, were correlated with disease severity (7). In another instance, extensive superficial WM changes were observed in patients with anti-NMDAR encephalitis and was correlated with cognitive impairment (8). In addition, Liang et al. revealed that patients with anti-NMDAR encephalitis had reduced FA values in the left middle cerebellar peduncle, right middle temporal gyrus, and precuneus, and increased mean diffusivity (MD) values in the middle temporal gyrus and frontal lobe compared with control subjects, with the WM alterations in the right precuneus correlated with cognitive impairment (9). These studies imply WM alterations may correlate with clinical symptoms or disease severity. However, studies on WM impairment of anti-NMDAR encephalitis are scarce. Therefore, more in-depth research is necessitated to explore WM alterations in patients with anti-NMDAR encephalitis.

Multivariate pattern analysis (MVPA) is a machine-learning-based analytical method for imaging studies. It can simultaneously analyze the spatial patterns of multiple voxel signal formation, utilize more data for more sensitive analysis, and apply these data to classify individuals into distinct categories with improved classification efficacy (10, 11). To date, MVPA has been increasingly utilized for the study of neuropsychiatric disorders such as epilepsy and Alzheimer's disease (12, 13). Nevertheless, the application of MVPA to anti-NMDAR encephalitis studies is extremely uncommon. The early diagnosis and treatment of patients with anti-NMDAR encephalitis are of great significance for their prognosis (14). However, its definitive diagnosis currently relies on antibody detection in cerebrospinal fluid and serum which is time-consuming and often leads to delayed diagnosis. In addition, antibody detection is limited in its prognostic capability for anti-NMDAR encephalitis (15). Therefore, further reliable biomarkers are warranted besides antibody detection for the diagnosis and prognostic assessment of anti-NMDAR encephalitis.

This study aimed to explore WM microstructural alterations in patients with anti-NMDA receptor encephalitis and identify potential novel imaging biomarkers using the MVPA approach.

## Materials and methods

### Participants

Thirty-two patients with anti-NMDAR encephalitis attending the Department of Neurology at The First Affiliated Hospital of Guangxi Medical University from January 2019 to December 2021, and 26 age- and gender- matched healthy controls were recruited in this study. Only patients meeting the following criteria were enrolled in the case group: 1. Patients diagnosed with anti-NMDAR encephalitis according to the Graus diagnostic criteria (2): (1) one or more of the following characteristic symptoms (psychiatric symptoms or cognitive dysfunction; language dysfunction; seizures; movement disorders or involuntary movement; consciousness disturbance; dysautonomia or central hypoventilation); (2) positive anti-NMDAR antibodies (IgG) in the cerebrospinal fluid and/or serum; (2) Right-handedness. Individuals were excluded from this study if they suffered from severe internal diseases and other neurological disorders. The severity of the patients' conditions was assessed using the Glasgow Coma Scale (GCS) and modified Rankin Scale (mRS) scores by two experienced neurologists at study time point, as presented in Table 1. None of the controls suffered from neurological and psychiatric disorders or severe systemic diseases, and all were right-handed. Age and gender differences were estimated using two-sample *t*-test and Chi-squared test *via* SPSS 22.0, respectively. The significance level for group differences was set to *P* < 0.05.

**Table 1:** General information and clinical features of patients with anti-NMDAR encephalitis.

	**Gender**	**Age**	**IgG NMDAR antibodies**		**GCS at the study time point**	**mRS at the study time point**	**Immunotherapy at the study time point**	**Symptoms**
			**Anti–NMDAR Antibodies(CSF)**	**Anti–NMDAR Antibodies(Serum)**				
1	F	29	1:32	—	12	4	Glucocorticoids, IVIG	Psychiatric symptoms, seizure, decrease in level of consciousness
2	F	33	1:10	1:10	5	5	Glucocorticoids	Psychiatric symptoms, seizure, memory impairment, decrease in the level of consciousness, central hypoventilation
3	F	56	1:10	1:10	10	4	Glucocorticoids, IVIG	Psychiatric symptoms, seizure, decrease in level of consciousness
4	F	25	1:32	1:10	15	2	Glucocorticoids, IVIG	Psychiatric symptoms, seizure, memory impairment
5	F	17	1:32	1:32	15	3	Glucocorticoids, IVIG, PE	Psychiatric symptoms, seizure
6	M	17	1:10	1:10	15	2	Glucocorticoids	Psychiatric symptoms, seizure, involuntary movement, cognitive deficits, memory dysfunction
7	F	16	1:10	1:10	13	3	Glucocorticoids, IVIG	Psychiatric symptoms, language dysfunction, sleep disorders
8	M	37	1:3.2	–	14	4	Glucocorticoids	Seizure, language dysfunction
9	M	44	1:1	–	12	5	Glucocorticoids, IVIG, PE	Psychiatric symptoms, seizure, dysautonomia
10	F	28	1:1	–	5	5	Glucocorticoids	Psychiatric symptoms, seizure, decrease in level of consciousness, memory impairment
11	M	20	+	+	5	5	Glucocorticoids, IVIG, PE	Psychiatric symptoms, seizure, decrease in level of consciousness, central hypoventilation
12	M	17	1:32	–	15	1	Glucocorticoids, IVIG	Psychiatric symptoms, seizure, memory impairment
13	F	21	1:10	1:100	13	4	Glucocorticoids, IVIG	Psychiatric symptoms, seizure
14	F	22	+	+	15	2	Glucocorticoids	Psychiatric symptoms, seizure, involuntary movement, memory impairment
15	M	25	1:32	1:10	11	4	Glucocorticoids	Psychiatric symptoms, seizure
16	F	32	+	–	5	5	Glucocorticoids, IVIG, PE	Psychiatric symptoms, seizure, involuntary movement, language dysfunction, decreased level of consciousness, central hypoventilation
17	F	16	1:10	–	5	5	Glucocorticoids, IVIG, PE	Psychiatric symptoms, seizure, language dysfunction, decrease in level of consciousness, central hypoventilation
18	M	19	1:32	1:320	15	4	Glucocorticoids, IVIG, PE	Psychiatric symptoms, seizure, involuntary movement, decrease in level of consciousness, memory impairment
19	F	49	1:3.2	–	14	2	Glucocorticoids, IVIG	Psychiatric symptoms, seizure
20	M	27	1:32	1:10	15	1	Glucocorticoids, IVIG	Psychiatric symptoms, seizure, memory impairment
21	M	23	1:1	–	5	5	Glucocorticoids, IVIG, PE	Psychiatric symptoms, seizure, decrease in level of consciousness, central hypoventilation
22	M	30	1:100	1:10	14	2	Glucocorticoids	Psychiatric symptoms, seizure, language dysfunction, memory impairment
23	M	30	1:32	1:100	14	4	Glucocorticoids	Psychiatric symptoms, seizure, decrease in level of consciousness
24	F	27	1:100	–	13	4	Glucocorticoids	Psychiatric symptoms, seizure, decrease in level of consciousness, memory impairment
25	M	47	1:10	–	14	3	Glucocorticoids, IVIG	Psychiatric symptoms, seizure, memory impairment
26	M	35	1:10	+	14	2	Glucocorticoids, IVIG, PE	Psychiatric symptoms, seizure, language dysfunction, involuntary movement, memory impairment
27	M	29	+	–	15	2	Glucocorticoids	Seizure
28	F	23	+	–	13	2	Glucocorticoids	Psychiatric symptoms, seizure, decrease in level of consciousness.
29	F	31	+	–	15	2	Glucocorticoids	Psychiatric symptoms, seizure
30	M	20	+	–	5	5	Glucocorticoids, PE	Psychiatric symptoms, seizure, decrease in level of consciousness, central hypoventilation
31	M	27	1:320	1:100	5	5	Glucocorticoids, IVIG	Psychiatric symptoms, seizure, language dysfunction, movement disorders, decrease in the level of consciousness, central hypoventilation, memory impairment
32	F	23	+	–	15	1	Glucocorticoids	Psychiatric symptoms, seizure, memory impairment

NMDAR, N-methyl-d-aspartate receptor; IgG, immunoglobulin G; GCS, Glasgow Coma Scale; mRS, modified Rankin Scale; M, male; F, female; IVIG, intravenous gamma globulin; PE, plasma exchange.

Ethical approval was granted by the local ethics committees of The First Affiliated Hospital of Guangxi Medical University in Guangxi Province, China, with written informed consent obtained from all participants.

### MRI data acquisition

MRI data were acquired from a Philips Achieva 3.0-T scanner with a 12 channels standard head coil. Foam padding was used to reduce the participants' head motion. DTI images were obtained with 32 gradient directions using a diffusion-weighted echo-planar imaging (DW-EPI) sequence with the following scanning parameters: TR = 1,000 ms, TE = 15 ms, flip angle = 90°, slice thickness = 2 mm without a gap, FOV = 224 mm × 224 mm, matrix = 112 × 112, b-value = 1,000 s/mm^2^.

### Data processing

The pipeline toolbox PANDA (16) was employed to call the FMRIB Software Library (FSL) (17) for DTI image dataset preprocessing. The steps were as follows: extraction of b0 values, skull-stripping, head motion and eddy current correction, calculation of diffusion tensor, non-linear alignment of the tensor data to standard space, and creation of mean tensor images and WM tracts skeleton with the threshold set to 0.2. The FA, MD, radial diffusivity (RD), and axial diffusivity (AD) images for each subject were obtained by the above steps. Voxel-based statistical analyses of FA, MD, RD, and AD were performed using Tract-based spatial statistics (TBSS), with gender and age as covariates. Results were FWE-corrected (*p* < 0.01) using the threshold-free cluster enhancement (TFCE) non-parametric permutation test with 5,000 permutations.

Additionally, to verify the robustness of the results, an atlas-based analysis was conducted using the values calculated for each region from each subject, and the results were statistically analyzed using independent two-samples *t-*test with FDR correction (*p* < 0.05).

### Correlation analysis

To investigate whether WM alterations were correlated to mRS and GCS scores, a partial correlation analysis was used for those DTI parameters, with gender and age as covariates (*P* < 0.05).

### Multivariate pattern analysis

MVPA was performed *via* the PRoNTo Toolbox (http://www.mlnl.cs.ucl.ac.uk/pronto) to estimate potential WM areas for differentiating patients with anti-NMDAR encephalitis from control subjects based on FA and RD images. The main steps included (1) feature selection; (2) training the SVM classifier; (3) evaluating the classifier model; (4) calculating the weights of brain regions which contributed to the classification. In this study, a separating hyperplane that optimizes the separation between two classes is sought for by an SVM after first mapping the 3D image input data into a high-dimensional feature space. To validate SVM classifiers, a 10-fold cross-validation approach was used, with feature selection conducted on the training data each time to prevent circularity effects. In this work, one out of 10 participants were excluded from each category and the classifier was trained using the remaining participants. The removed subjects were then used to evaluate the classifier's ability to correctly categorize fresh instances. The above processes were performed for each fold in order to produce a relatively unbiased assessment of generalizability. Finally, the accuracy, sensitivity, and specificity were obtained based on the FA and RD maps, and a non-parametric permutation test (1,000 times) was applied to determine statistical significance. Besides, the receiver operating characteristic (ROC) curve and the area under the curve (AUC) were calculated to evaluate the performance of the classifier model. For feature extraction and selection, the JHU-ICBM-DTI-81 atlas was utilized in this study.

## Results

### Clinical features

No significant differences were observed between the two groups regarding gender and age (*p* > 0.05). The patients had a mean mRS score of 3.3 (1–5) and a mean GCS score of 11.6 (5–15). Psychiatric symptoms and seizures were present in 93.75 and 96.88% of patients, respectively. More information is provided in Table 1.

### WM alterations

**Figure 1:**
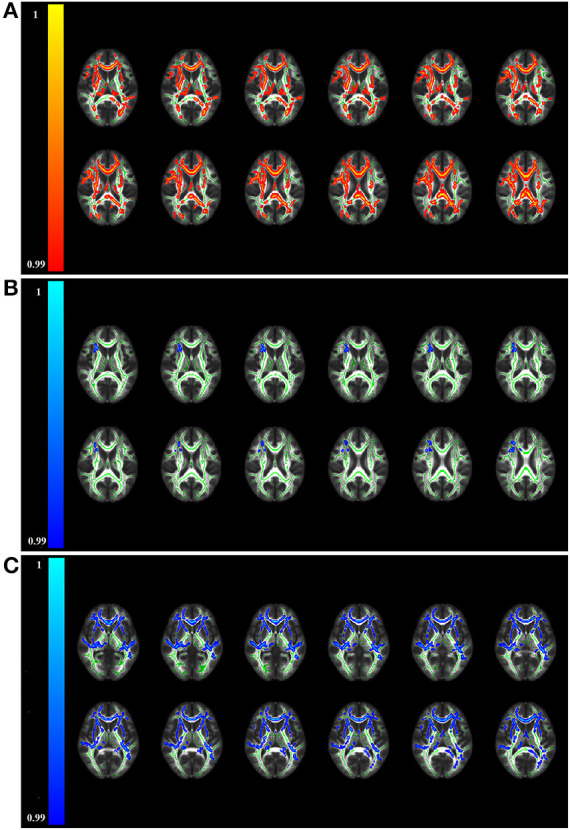
Brain regions displaying significantly altered fractional anisotropy **(A)**, mean diffusivity **(B)**, and radial diffusivity **(C)** in patients compared to controls by TBSS analysis (*p* < 0.01, FWE corrected). The red/yellow colors represent reduced FA in patients, while blue/light-blue colors represent increased MD and RD in patients relative to controls (color-coded according to the 1 - *p*-value). No significant differences in axial diffusivity were observed between the two groups. TBSS, tract-based spatial statistics.

**Figure 2:**
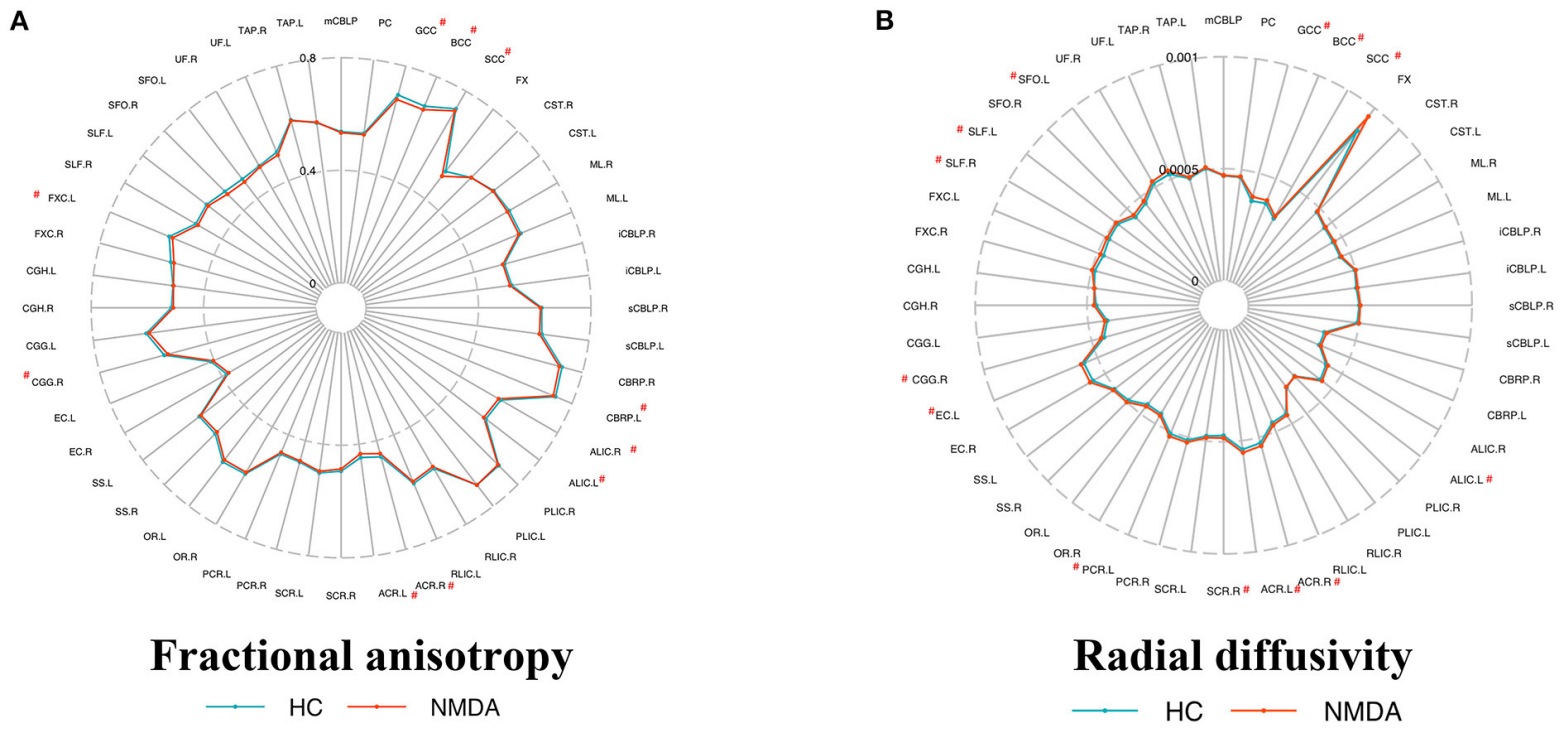
Metrics of the white matter in patients and controls by atlas-based analysis. Fractional anisotropy **(A)** and radial diffusivity **(B)** metrics of ICBM-DTI-81 WM label atlas, respectively. Atlas that shows a significant difference (*p* < 0.05 FDR correction) is marked with a “#” at the top corner. No significant differences in axial diffusivity and mean diffusivity were observed between the two groups. All abbreviations are listed in Supplementary Table 2.

TBSS analysis uncovered patients with anti-NMDAR encephalitis had significantly decreased FA compared to healthy controls (*p* < 0.01, FWE corrected), predominantly located in the corpus callosum, fornix, anterior limb of the internal capsule (ALIC), anterior corona radiata (ACR), retrolenticular part of the internal capsule (RLIC), superior corona radiata, and cingulum (Table 2 and Figure 1A). On the one hand, no areas with significantly increased FA were identified. On the other hand, mean diffusivity was elevated in the ALIC, ACR, body of the corpus callosum, and superior corona radiata (*p* < 0.01, FWE corrected) (Table 2 and Figure 1B). Moreover, RD was increased in the corpus callosum, ALIC, ACR, cingulum, and superior corona radiata (*p* < 0.01, FWE corrected) (Table 2 and Figure 1C). No regions with reduced MD and RD were noted. However, there was no between-group difference in AD. Atlas-based analysis revealed significantly decreased FA in the corpus callosum, cerebral peduncle, ALIC, ACR, cingulum, and fornix of patients with anti-NMDA receptor encephalitis (*p* < 0.05, FDR corrected) (Table 3 and Figure 2A). In addition, patients exhibited an extensive increased RD in the corpus callosum, ALIC, corona radiata, external capsule, cingulum, superior longitudinal fasciculus, and superior fronto-occipital fasciculus (*p* < 0.05, FDR corrected) (Table 3 and Figure 2B). However, no significantly between-group differences in AD and MD were noted.

**Table 2:** Clusters with a significant between-group difference by TBSS analysis.

**Regions**	**Abbreviation**	**Peak MNI coordinates**			P-value^*^	**Cluster size**
		**x**	**y**	**z**		
**FA**						
Genu of corpus callosum	GCC	−3	18	18	0.0006	25913^a^
Body of corpus callosum	BCC	−7	22	16	0.0006	25913^a^
Splenium of corpus callosum	SCC	−10	−28	26	0.005	25913^a^
Anterior limb of internal capsule R	ALIC.R	18	8	10	0.005	25913^a^
Anterior corona radiata R	ACR.R	26	28	7	0.005	25913^a^
Anterior corona radiata L	ACR.L	−15	34	−7	0.0006	25913^a^
Superior corona radiata R	SCR.R	24	3	19	0.005	25913^a^
Superior corona radiata L	SCR.L	−18	4	39	0.0006	25913^a^
Cingulum (cingulate gyrus) R	CGG.R	9	2	33	0.006	25913^a^
Cingulum (cingulate gyrus) L	CGG.L	−8	24	21	0.002	25913^a^
Cingulum (hippocampus) L	CGH.R	24	−28	−18	0.002	25913^a^
Retrolenticular part of internal capsule R	RLIC.R	32	−27	−1	0.007	1594^b^
Fornix R	FXC.R	32	−14	−12	0.008	1594^b^
**MD**						
Body of corpus callosum	BCC	12	15	24	0.009	68
Anterior limb of internal capsule R	ALIC.R	22	20	5	0.009	603^c^
Anterior corona radiata R	ACR.R	26	22	8	0.009	603^c^
Superior corona radiata R	SCR.R	26	−9	32	0.009	918
**RD**						
Genu of corpus callosum	GCC	−14	29	17	0.001	34706^d^
Body of corpus callosum	BCC	0	18	18	0.001	34706^d^
Splenium of corpus callosum	SCC	−18	−49	18	0.009	34706^d^
Anterior limb of internal capsule R	ALIC.R	20	8	11	0.002	34706^d^
Anterior corona radiata R	ACR.R	24	21	10	0.002	34706^d^
Anterior corona radiata L	ACR.L	−16	26	21	0.001	34706^d^
Superior corona radiata R	SCR.R	26	−11	32	0.001	34706^d^
Cingulum (cingulate gyrus) R	CGG.R	9	12	30	0.008	34706^d^

TBSS, tract-based spatial statistics; FA, fractional anisotropy; MD, mean diffusivity; RD, radial diffusivity;

* : FWE correction; a, b, c, and d indicate that these brain regions share a cluster.

**Table 3:** Regions with a significant between-group difference by atlas-based analysis.

**Region**	**Abbreviation**	***P*** **-value** ^*^	
		**FA**	**RD**
Genu of corpus callosum	GCC	0.011	0.021
Body of corpus callosum	BCC	0.011	0.022
Splenium of corpus callosum	SCC	0.036	0.046
Cerebral peduncle L	CBRP.L	0.047	ns
Anterior limb of internal capsule R	ALIC.R	0.036	ns
Anterior limb of internal capsule L	ALIC.L	0.036	0.022
Anterior corona radiata R	ACR.R	0.036	0.044
Anterior corona radiata L	ACR.L	0.035	0.022
Superior corona radiata R	SCR.R	ns	0.021
Posterior corona radiata L	PCR.L	ns	0.039
External capsule L	EC.L	ns	0.022
Cingulum (cingulate gyrus) R	CGG.R	0.036	0.044
Fornix R	FXC.R	0.047	0.049
Superior longitudinal fasciculus R	SLF.R	ns	0.044
Superior longitudinal fasciculus L	SLF.L	ns	0.044
Superior fronto-occipital fasciculus L	SFO.L	ns	0.046

* : FDR correction; ns, not significant.

### Correlation analysis

**Figure 3:**
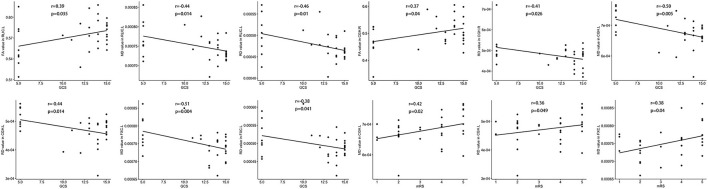
Correlation between white matter damage and GCS or mRS score in patients with anti-NMDAR encephalitis. FA, fractional anisotropy; MD, mean diffusivity; RD, radial diffusivity. All brain region abbreviations are listed in Supplementary Table 2.

The FA value of the left RLIC and right cingulum was positively correlated with the GCS score (Figure 3). In contrast, the MD value of the left RLIC, cingulum, and fornix was negatively correlated with the GCS score, whereas that of the left cingulum and fornix was positively correlated with the mRS score (Figure 3). The RD value of the left RLIC, fornix, and bilateral cingulum was negatively correlated with the GCS score, while that of the left cingulum was positively correlated with the mRS score (Figure 3).

### MVPA classification evaluation

**Figure 4:**
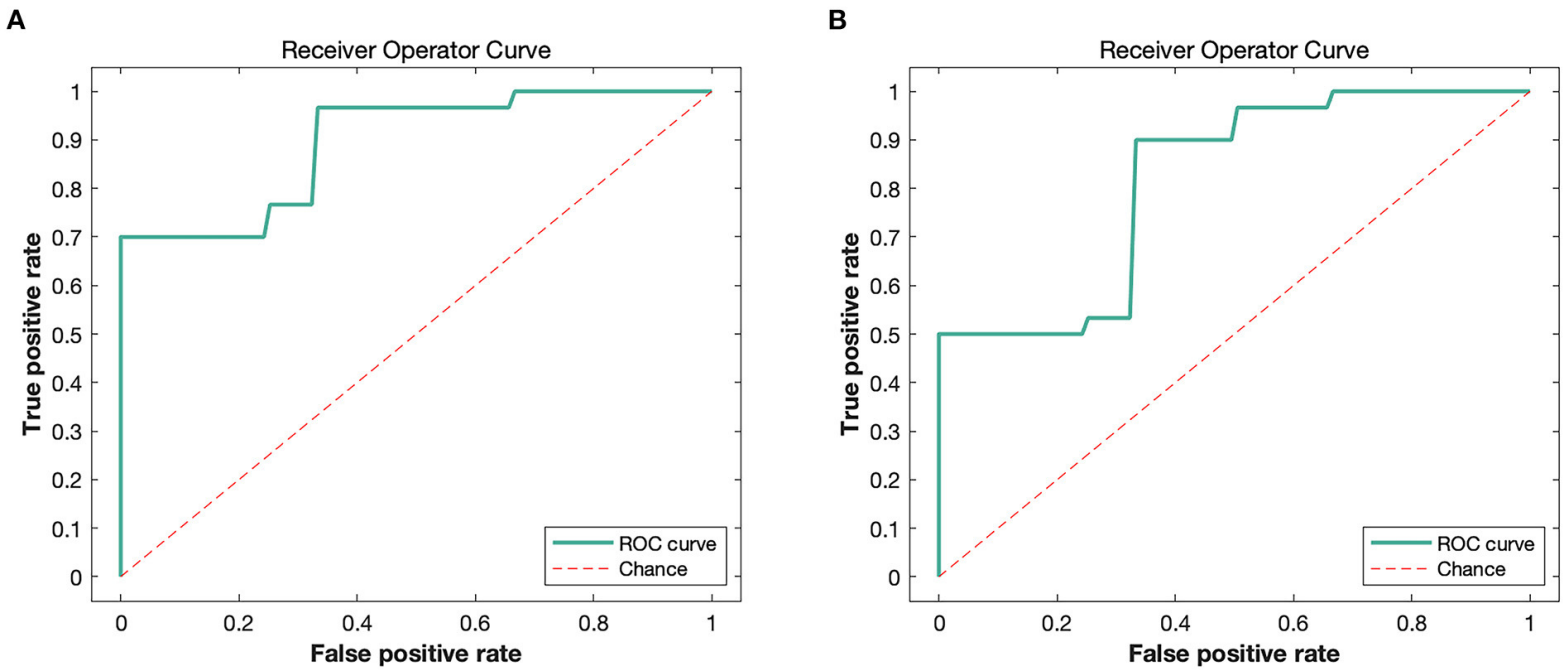
The ROC curves of classifier performance of FA-based **(A)** and RD-based **(B)**. FA, fractional anisotropy; RD, radial diffusivity.

**Figure 5:**
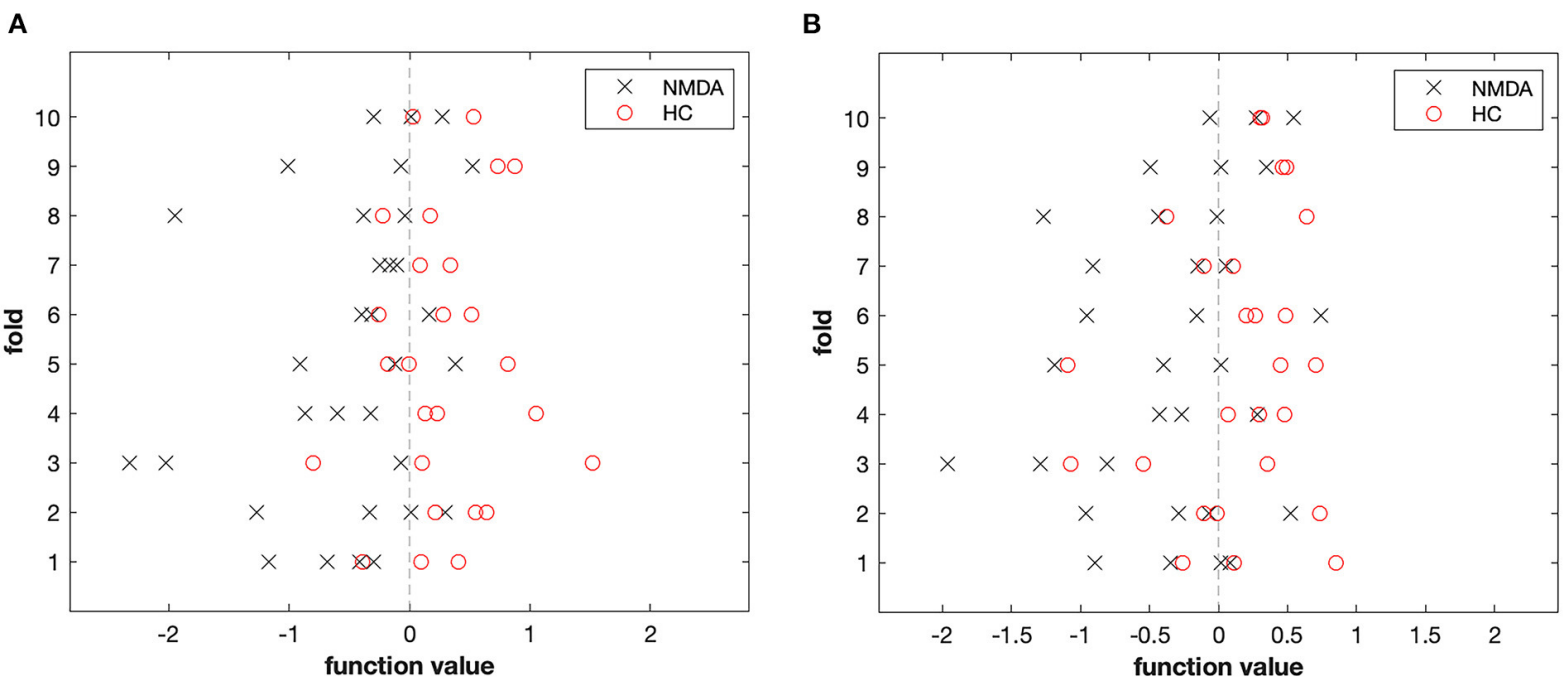
The result of the MVPA classification between 32 patients and 26 controls based on FA **(A)** and RD **(B)** derived from DTI data.

Patients with anti-NMDAR encephalitis and control subjects were discriminated against based on FA and RD images. The accuracy (permutation *p* = 0.001, 1,000 times), sensitivity, and specificity of FA-based classification were each 78.33%, and the AUC was 0.89 (Table 4 and Figure 4A). Regarding RD, accuracy was 67.71% (permutation *p* = 0.03, 1,000 times), with a specificity of 70%, a sensitivity of 65.83%, and an AUC of 0.81 (Table 4 and Figure 4B). Figure 5 illustrates the classification results based on FA and RD. The most informative regions for discrimination based on FA were in the middle cerebellar peduncle, body of corpus callosum, posterior thalamic radiation, and ACR, whereas based on RD, they were the genu of the corpus callosum, posterior limb of the internal capsule, superior cerebellar peduncle, and ALIC (More details are presented in Supplementary Table 1).

**Table 4:** MVPA classification results (10-fold cross-validation).

	**Accuracy**	**Sensitivity**	**Specificity**	**AUC**	**Permutation**
	**(%)**	**(%)**	**(%)**		***P-*values**
FA	78.33	78.33	78.33	0.89	0.001
RD	67.71	65.83	70	0.81	0.03

## Discussion

Anti-NMDAR encephalitis is one of the most commonly encountered autoimmune encephalitides, yet MRI studies on anti-NMDAR encephalitis are scarce. Herein, WM changes of a well-matched group of patients with anti-NMDA receptor encephalitis were compared to healthy controls using unbiased automated TBSS and atlas-based analytical approaches.

Our study revealed widespread alterations in the WM of patients with anti-NMDAR encephalitis. Compared with controls, patients exhibited significantly decreased FA in the corpus callosum, fornix, ALIC, ACR, and cingulum. In comparison, MD was elevated in the ACR, body of the corpus callosum, and superior corona radiata. On the other hand, RD was significantly increased in the corpus callosum, ALIC, cingulum, and ACR, while no significant changes were found in AD. This outcome is consistent with previous findings on WM in patients with anti-NMDR encephalitis (7, 18).

DTI is a technique that can measure the diffusion of water molecules in brain tissue. In this study, four diffusion metrics, FA, MD, AD, and RD, were used to fully assess the microstructural integrity of WM in patients. FA, the most frequently used parameter, describes the differences between diffusion metrics in three different diffusion directions, related to axon number, axon density, axon diameter, and myelination (19, 20). MD is an average measurement of diffusion metrics in the three directions. AD is related to axon diameter, number, and density, whereas RD is mainly influenced by the loss of myelin sheaths.

In the present study, widespread WM alterations were found in patients, as evidenced by decreased FA and increased MD and RD, without significant changes in AD. This outcome is in line with a previous study (7). This pattern of abnormal WM microstructural alterations is hypothesized to be the result of demyelination rather than axonal damage (21).

Earlier studies have reported that the low expression of NMDAR on synaptic membranes in patients with anti-NMDAR encephalitis is caused by anti-NMDAR antibodies that disrupt the interaction of the receptors with their anchoring proteins, thereby leading to the internalization of NMDA membrane receptors, but this specific alteration does not affect the number of excitatory synapses (22–24). This means that anti-NMDAR antibodies may not influence the number and density of axons. In addition, recent studies have postulated that NMDAR play a crucial role in remyelination (25, 26). Myelin sheaths originate from oligodendrocyte precursor cells, and glutamate release from axons triggered by neuronal activity during the early stages of myelin formation is instructive for myelin induction (27–29). However, internalization of NMDAR in patients with anti-NMDAR encephalitis inactivates NMDA receptor-rich GABAergic neurons, resulting in an increase in extracellular glutamate from neurons. These frameworks may explain why the decrease in FA in patients is primarily attributed to an increase in RD rather than a decrease in AD. Therefore, low expression of NMDAR may influence remyelination, leading to a decline in myelin integrity. The WM damage observed may be attributed to demyelination caused by the autoantibody-targeted attack on NMDAR in oligodendrocytes.

Dalmau et al. described that 77% of patients with anti-NMDAR encephalitis develop a variety of psychiatric symptoms (1). In the present study, 93.75% of the patients displayed psychiatric behavioral abnormalities. Damage to WM microstructures may impact neuronal activity in brain regions connected by fiber tracts, leading to various clinical symptoms. Clinically, acute symptoms in patients with anti-NMDAR encephalitis are similar to those of schizophrenia, and NMDAR hypofunction is thought to be a central pathophysiological mechanism for schizophrenia-like symptoms (30–32). The NMDAR antagonist ketamine induces symptoms common to both disorders, such as cognitive dysfunction, memory impairment, and executive dysfunction (33, 34). Furthermore, we found that WM dispersion parameters in both anti-NMDAR encephalitis and schizophrenia exhibited increased RD and no change in AD (35–37). This outcome indicates that the integrity of myelin is compromised in both patients with schizophrenia and anti-NMDAR encephalitis and that these impairments may account for the psychiatric symptoms.

Previous studies on schizophrenia have evidenced that FA is diminished in the ACR, corpus callosum, fornix, and ALIC in schizophrenic patients, and our findings are consistent with these prior studies (38–40). The corpus callosum is the largest bridge connecting the bilateral cerebral hemispheres and plays a crucial role in transmitting information between the cerebral hemispheres. The genu of the corpus callosum is connected to the medial frontal lobe, an important brain region involved in memory, emotion, and decision-making behavior. Injury to the WM of the corpus callosum may substantially impact brain information transfer activities and trigger psychiatric disorders. Previous studies on both first-episode and chronic schizophrenia have reported abnormal WM changes in the corpus callosum of patients (40–42), which are associated with psychiatric symptoms (43). Damage to the ALIC, which contains the interconnecting fibers of the thalamus and prefrontal cortex, may result in reduced connectivity between the thalamus and prefrontal cortex and disruption of the limbic system's sensory feedback pathways (44). WM integrity decline in the ALIC may also underlie certain neuropsychological disorders observed in schizophrenia (40, 45).

The fornix is the major output nerve fiber of the hippocampus, and damage to it may cause memory impairment and other neuropsychiatric symptoms. The ACR is a bundle of nerve fibers that connects the anterior cingulate cortex to other brain regions (46). Reduced anterior cingulum activation is associated with attention deficit disorder, schizophrenia, and other psychiatric disorders (47, 48). Previous studies have indicated widespread WM change in patients with anti-NMDAR encephalitis, particularly in the cingulum (7). The cingulum (hippocampus) is the major input nerve fiber of the hippocampus, connecting the ventromedial prefrontal cortex, temporoparietal cortex, cingulate gyrus, and parahippocampal gyrus, and forming a neurotransmission pathway associated with higher neural activities such as learning, memory, and emotion, referred to as the hippocampal loop or Papez circuit. WM damage in the fornix, cingulum (cingulate gyrus), and cingulum (hippocampus) may impede or even disrupt the transfer of neuronal information in this pathway, leading to disruption of the hippocampal connections with other brain structures, resulting in memory and executive function impairment. In addition, previous studies have demonstrated that medial temporal lobe epilepsy patients also exhibited reduced FA in the fornix, cingulum, external capsule, and corpus callosum, which may be the result of recurrent seizures (49–51). Herein, 96.88% of patients experienced seizures. Therefore, we hypothesized that the loss of myelin sheaths in these areas due to anti-NMDA receptor antibodies may cause white matter damage, leading to dysfunction of these areas and then resulting in schizophrenia-like symptoms and seizures. However, there is no research to confirm it, and further research is needed to confirm our speculation.

Correlation analysis identified that WM damage in the cingulum (hippocampus), RLIC, and fornix were correlated with disease severity. Previous studies have uncovered that disease severity is significantly correlated with WM damage (7, 8), consistent with our findings. The cingulate (hippocampus) and the fornix are the major input and output nerve fibers of the hippocampus, respectively. Moreover, they are essential components of the Papez circuit, which play an instrumental role in cognition, emotion, and memory. It has been shown that language and memory impairment are correlated with left hippocampal atrophy and reduced functional connectivity between the hippocampus and medial temporal lobe network are correlated with memory impairment in patients with anti-NMDAR encephalitis (7, 52).

MVPA is an MRI data analysis method based on pattern recognition theory and machine learning. In this study, FA and RD were used as features for machine learning to discriminate patients with anti-NMDAR encephalitis from control subjects to discover potential imaging biomarkers. FA proved superior, with a classification accuracy of 78.33%, comparable to the ROC analysis of DTI-derived FA in the previous study (7). The most informative regions for distinguishing patients with anti-NMDAR encephalitis from controls for FA were the middle cerebellar peduncle, body corpus callosum, ACR, and posterior thalamic radiation, which is in line with the results of previous machine learning studies (18) and is also supported by the group-level TBSS results in the present study. Surprisingly, the middle cerebellar peduncle had the largest weight in the classification, yet we did not find between-group differences, which may be owing to the machine learning algorithm taking into account the connectivity and correlation between regions with a different focus than the group comparison method (53).

There are several limitations herein. First, the sample size of this study was limited, and multiple comparison correction methods were performed to evaluate the diffusion metrics. Therefore, alterations in WM observed in patients with anti-NMDAR encephalitis should be validated in a larger sample size. Second, considering this was a cross-sectional study, we cannot rule out the impact of disease progression or treatment on our findings. Lastly, no correlation analysis was conducted between diffusion metrics and neuropsychological testing in this study.

In conclusion, the present study identified widespread WM abnormalities in patients with anti-NMDAR encephalitis based on TBSS and atlas-based analyses. Some of these abnormal WM alterations were correlated with disease severity. The results of the DTI image analysis may be helpful for improved understanding of the clinico-radiological paradox in anti-NMDAR encephalitis. The combination of TBSS and MVPA may provide more specific and complementary information, which may help to improve our understanding of the underlying pathophysiological mechanisms of anti-NMDAR encephalitis at the group and individual levels.

## Data availability statement

The original contributions presented in the study are included in the article/Supplementary material, further inquiries can be directed to the corresponding author.

## Ethics statement

The studies involving human participants were reviewed and approved by the local Ethics Committees of The First Affiliated Hospital of Guangxi Medical University. The patients/participants provided their written informed consent to participate in this study.

## Author contributions

SY and YW: study concept, design, and drafting of the manuscript. All authors: data acquisition, analysis, interpretation, and critical review of manuscript. All authors contributed to the article and approved the submitted version.

## Funding

This work was supported by grants from Natural Science Foundation of Guangxi Province (2020GXNSFDA297012).

## Conflict of interest

The authors declare that the research was conducted in the absence of any commercial or financial relationships that could be construed as a potential conflict of interest.

## Publisher's note

All claims expressed in this article are solely those of the authors and do not necessarily represent those of their affiliated organizations, or those of the publisher, the editors and the reviewers. Any product that may be evaluated in this article, or claim that may be made by its manufacturer, is not guaranteed or endorsed by the publisher.
