# Supplementary material for: White matter abnormalities and multivariate pattern analysis in anti-NMDA receptor encephalitis
Source: Front Psychiatry. 2022 Sep 23;13:997758. doi: 10.3389/fpsyt.2022.997758 (PMC9537694; doi:10.3389/fpsyt.2022.997758)
Supplement: Supplementary file 1 [file Table_1.DOCX]

Table S1

Brain regions contributing to classification between the two groups

|  | **Region** | **Weight (%)** | **Cluster size (vox)** |
| --- | --- | --- | --- |
| FA | Middle cerebellar peduncle | 61.49 | 15644 |
|  | Body of corpus callosum | 30.15 | 13711 |
|  | Posterior thalamic radiation L | 4.87 | 3978 |
|  | Anterior corona radiata L | 3.49 | 6852 |
|  |  |  |  |
| RD |  |  |  |
|  | Genu of corpus callosum | 26.71 | 8851 |
|  | Posterior limb of internal capsule L | 15.14 | 3752 |
|  | Superior cerebellar peduncle R | 13.21 | 992 |
|  | Anterior limb of internal capsule R | 7.05 | 3138 |
|  | Inferior cerebellar peduncle L | 6.7 | 839 |
|  | Anterior limb of internal capsule L | 6.45 | 3018 |
|  | Superior fronto-occipital fasciculus L | 3.88 | 507 |
|  | Posterior fronto-occipital fasciculus R | 3.65 | 3754 |
|  | Uncinate fasciculus L | 3.62 | 376 |
|  | Inferior cerebellar peduncle R | 3.16 | 839 |
|  | Fornix R | 1.63 | 1124 |
|  | Anterior corona radiata L | 1.54 | 6852 |
|  | Superior corona radiata R | 1.34 | 7500 |
|  | Tapetum R | 1.34 | 596 |
|  | Posterior corona radiata L | 1.31 | 3714 |
|  | Middle cerebellar peduncle | 1.22 | 15644 |
|  | Retrolenticular part of internal capsule L | 0.72 | 2469 |
|  | Body of corpus callosum | 0.3 | 13711 |
|  | Cingulum (cingulate gyrus) R | 0.3 | 2342 |
|  | Superior longitudinal fasciculus L | 0.29 | 6605 |
|  | External capsule L | 0.25 | 5587 |
|  | Cingulum (hippocampus) R | 0.09 | 1236 |
|  | Superior corona radiata L | 0.06 | 7508 |
|  | Splenium of the corpus callosum | 0.04 | 12729 |
