# Supplementary material for: White matter abnormalities and multivariate pattern analysis in anti-NMDA receptor encephalitis
Source: Front Psychiatry. 2022 Sep 23;13:997758. doi: 10.3389/fpsyt.2022.997758 (PMC9537694; doi:10.3389/fpsyt.2022.997758)
Supplement: Supplementary file 2 [file Table_2.DOCX]

Table S2

Names and indices of the JHU-ICBM-DTI-81 white matter atlas

| Region | Abbreviations | Index |
| --- | --- | --- |
| Middle cerebellar peduncle | mCBLP | 1 |
| Pontine crossing tract | PC | 2 |
| Genu corpus callosum | GCC | 3 |
| Body corpus callosum | BCC | 4 |
| Splenium corpus callosum | SCC | 5 |
| Fornix | FX | 6 |
| Corticospinal tract R | CST.R | 7 |
| Corticospinal tract L | CST.L | 8 |
| Medial lemniscus R | ML.R | 9 |
| Medial lemniscus L | ML.L | 10 |
| Inferior cerebellar peduncle R | iCBLP.R | 11 |
| Inferior cerebellar peduncle L | iCBLP.L | 12 |
| Superior cerebellar peduncle R | sCBLP.R | 13 |
| Superior cerebellar peduncle L | sCBLP.L | 14 |
| Cerebral peduncle R | CBRP.R | 15 |
| Cerebral peduncle L | CBRP.L | 16 |
| Anterior limb of internal capsule R | ALIC.R | 17 |
| Anterior limb of internal capsule L | ALIC.L | 18 |
| Posterior limb of internal capsule R | PLIC.R | 19 |
| Posterior limb of internal capsule L | PLIC.L | 20 |
| Retrolenticular part of internal capsule R | RLIC.R | 21 |
| Retrolenticular part of internal capsule L | RLIC.L | 22 |
| Anterior corona radiata R | ACR.R | 23 |
| Anterior corona radiata L | ACR.L | 24 |
| Superior corona radiata R | SCR.R | 25 |
| Superior corona radiata L | SCR.L | 26 |
| Posterior corona radiata R | PCR.R | 27 |
| Posterior corona radiata L | PCR.L | 28 |
| Posterior thalamic radiation R | OR.R | 29 |
| Posterior thalamic radiation L | OR.L | 30 |
| Sagittal stratum R | SS.R | 31 |
| Sagittal stratum L | SS.L | 32 |
| External capsule R | EC.R | 33 |
| External capsule L | EC.L | 34 |
| Cingulum (cingulate gyrus) R | CGG.R | 35 |
| Cingulum (cingulate gyrus) L | CGG.L | 36 |
| Cingulum (hippocampus) R | CGH.R | 37 |
| Cingulum (hippocampus) L | CGH.L | 38 |
| Fornix (cres) R | FXC.R | 39 |
| Fornix (cres) R | FXC.L | 40 |
| Superior longitudinal fasciculus R | SLF.R | 41 |
| Superior longitudinal fasciculus L | SLF.L | 42 |
| Superior fronto-occipital fasciculus R | SFO.R | 43 |
| Superior fronto-occipital fasciculus L | SFO.L | 44 |
| Uncinate fasciculus R | UF.R | 45 |
| Uncinate fasciculus L | UF.L | 46 |
| Tapetum R | TAP.R | 47 |
| Tapetum L | TAP.L | 48 |
